# Erratum to: BiOsimilaRs in the management of anaemia secondary to chemotherapy in HaEmatology and Oncology: results of the ORHEO observational study

**DOI:** 10.1186/1471-2407-14-720

**Published:** 2014-09-26

**Authors:** Mauricette Michallet, Elisabeth Luporsi, Pierre Soubeyran, Nadia Ali Amar, Vincent Boulanger, Miguel Carreiro, Louis-Marie Dourthe, Jean-Luc Labourey, Daniel Lepille, Frédéric Maloisel, Jean-Loup Mouysset, Sophie Nahon, Bérengère Narciso, Pierre Nouyrigat, Raouf Radji, Nacéra Sakek, Hélène Albrand, The ORHEO study group

**Affiliations:** Centre Hospitalier Lyon Sud, Lyon, France; Centre Alexis Vautrin, Vandoeuvre-les-Nancy, France; Institut Bergonie, Bordeaux, France; Centre Hospitalier de Troyes, Troyes, France; Centre Hospitalier de Carcassonne, Carcassonne, France; Centre Hospitalier de Montauban, Montauban, France; Clinique Sainte Anne, Strasbourg, France; Polyclinique Montréal, Carcassonne, France; Clinique Pasteur, Evreux, France; Clinique Rambot-Provencale, Aix en Provence, France; Centre Hospitalier d’Aix en Provence, Aix en Provence, France; Centre Hospitalier Régional Universitaire de Tours, Tours, France; Clinique du Cap d’Or, La Seyne sur Mer, France; Centre Frédéric JOLLIOT, Rouen, France; Centre Hospitalier de Montbéliard, Montbeliard, France; Laboratoire HOSPIRA France, MeudonLa Foret, France

## Correction

After publication of this Research Article [1], we noted errors in Table 1, and in the rate of thromboembolic events (page 7). Specifically, in Table 1 the percentage of female and male patients were incorrectly given as 7% and 3% respectively. The correct figures are 48.6% female and 51.4% male (please see a corrected version of Table 1 below, and the Additional file 1). The correct rate of thromboembolic events is 3.49% (originally stated as 3.55%).

**Table 1:** **Patient demographics**

Characteristic	Value
Age, mean ± SD (years)	66.49 ± 11.77
Patients with solid tumours, mean (years)	65.76
Patients with lymphoma, mean (years)	68.59
Patients with myeloma, mean (years)	70.65
Female (%)	48.6
Male (%)	51.4
Hb, mean ± SD (g/dL)	9.59 ± 0.88
sBP, mean ± SD (mmHg)	125.75 ± 14.48
dBP, mean ± SD (mmHg)	74.18 ± 9.79
Malignancies, n (%)	2310 (100)
Solid tumours, n (%)	1838 (79.57)
Lymphoma, n (%)	301 (13.03)
Myeloma, n (%)	171 (7.40)
Height, mean ± SD (cm)	166.35 ± 8.72
Weight, mean ± SD (kg)	67.46 ± 14.23
BMI, mean ± SD (kg/m^2^)	24.33 ± 4.62

*BMI* body mass index, *dBP* diastolic blood pressure, *Hb* haemoglobin, *sBP* systolic blood pressure, *SD* standard deviation.

## Electronic supplementary material

Additional file 1: **Corrected version of article** ***BMC Cancer*** **14:503.** (PDF 433 KB)
