# Supplementary material for: Erratum to: BiOsimilaRs in the management of anaemia secondary to chemotherapy in HaEmatology and Oncology: results of the ORHEO observational study
Source: BMC Cancer. 2014 Sep 26;14:720. doi: 10.1186/1471-2407-14-720 (PMC4464620; doi:10.1186/1471-2407-14-720)
Supplement: Supplementary file 1 — Additional file 1: Corrected version of article BMC Cancer 14:503. (PDF 433 KB) [file 12885_2014_4891_MOESM1_ESM.pdf]

Michallet et al. BMC Cancer 2014, 14:503
http://www.biomedcentral.com/1471-2407/14/503

BMC Cancer

RESEARCH ARTICLE Open Access

BiOsimilaRs in the management of anaemia secondary to chemotherapy in HaEmatology and Oncology: results of the ORHEO observational study

Mauricette Michallet[1*], Elisabeth Luporsi[2], Pierre Soubeyran[3], Nadia Ali Amar[4], Vincent Boulanger[5], Miguel Carreiro[6], Louis-Marie Dourthe[7], Jean-Luc Labourey[5,8], Daniel Lepille[9], Frédéric Maloisel[7], Jean-Loup Mouysset[10], Sophie Nahon[11], Bérengère Narciso[12], Pierre Nouyrigat[13], Raouf Radji[14], Nacéra Sakek[15], Hélène Albrand[16] and The ORHEO study group

Abstract

Background: The approval of epoetin biosimilars in the European Union requires extensive scientific evaluation and stringent regulatory procedures, including post-marketing studies. The ORHEO (place of biOsimilaRs in the therapeutic management of anaemia secondary to chemotherapy in HaEmatology and Oncology) study was an observational, longitudinal, multicentre study performed in France to evaluate the efficacy and safety of biosimilar epoetins for the treatment of chemotherapy-induced anaemia (CIA) in the clinical setting.

Methods: Patients >18 years with CIA (haemoglobin [Hb] <11 g/dL) in association with solid tumours, lymphoma or myeloma and eligible for treatment with an epoetin biosimilar were included in this study. Patient characteristics were recorded at baseline along with anaemia-related information, such as observed and target Hb (as chosen by the treating clinician), brand and dose of epoetin biosimilar prescribed, and details of any other treatments. Patients were then followed-up at 3 and 6 months. The primary endpoint was Hb response (defined as Hb reaching ≥10 g/dL, an increase of Hb ≥1 g/dL since inclusion visit or reaching physician-defined target Hb, with no blood transfusions in the 3 weeks prior to measurement). Other endpoints included adverse events, achievement of target Hb and associated treatments.

Results: Overall, 2333 patients >18 years (mean age 66.5 years) with CIA (haemoglobin [Hb] <11 g/dL) in association with solid tumours, lymphoma or myeloma and eligible for biosimilar epoetin treatment were included. 99.9% of patients received epoetin zeta (median dose 30,000 IU/week). Mean baseline Hb was 9.61 g/dL, with 35.6% of patients having moderate anaemia (Hb 8–9.5 g/dL). Hb response was achieved in 81.6% and 86.5% of patients at 3 and 6 months, respectively. Overall mean change in Hb level was 1.52 ± 1.61 and 1.72 ± 1.61 g/dL at 3 and 6 months, respectively. Transfusion and thromboembolic event rates were 9.4% and 2.4% at 3 months, and 5.8% and 1.5% at 6 months, respectively.

Conclusions: Epoetin zeta was effective and well tolerated in the management of CIA in patients with solid tumours, lymphoma and myeloma.

Trial registration: Trial registration number: NCT02140736 (date of registration: 14 May 2014).

Keywords: Epoetin, ESA, Anaemia, Chemotherapy, Biosimilar

* Correspondence: Mauricette.michallet@chu-lyon.fr
[1]Service d'Hématologie, Pavillon Marcel Bérard 1G, Centre Hospitalier Lyon Sud, 165 Chemin du Grand Revoyet, Lyon- Pierre Bénite 69495, France
Full list of author information is available at the end of the article

BioMed Central

© 2014 Michallet et al.; licensee BioMed Central Ltd. This is an Open Access article distributed under the terms of the Creative Commons Attribution License (http://creativecommons.org/licenses/by/4.0), which permits unrestricted use, distribution, and reproduction in any medium, provided the original work is properly credited. The Creative Commons Public Domain Dedication waiver (http://creativecommons.org/publicdomain/zero/1.0/) applies to the data made available in this article, unless otherwise stated.

Background

Supportive therapy for patients undergoing chemotherapy can be of great benefit in alleviating undesirable consequences of this specific treatment. One such consequence is chemotherapy-induced anaemia (CIA), a common complication of myelosuppressive chemotherapy across a large range of cancer types. According to the European Cancer Anaemia Survey (ECAS), 67.0% of cancer patients were anaemic (haemoglobin [Hb] levels of <12 g/dL) during a 6-month period [1]. Patients presenting with CIA often suffer from fatigue and impaired quality of life (QoL) [2]. Successful Hb response through supportive therapy has been demonstrated to improve fatigue and QoL significantly in patients with CIA [2]. CIA is also independently associated with reduced survival rates for patients with solid tumours, lymphoma or myeloma [3], further highlighting the importance of providing appropriate supportive care.

Currently, anaemia treatment may involve red blood cell (RBC) transfusions, iron supplementation (in absolute or functionally iron-deficient anaemia) and erythropoiesis-stimulating agents (ESAs) [4]. RBC transfusions can provide a rapid, transient increase in Hb; however, as repeated transfusions are required to maintain Hb levels, this treatment is usually reserved for more severe cases of anaemia [5]. Furthermore, transfusions continue to be associated with risk of transfusion reactions or infections [6]. A systematic study of 125,223 patients undergoing major general surgery showed that transfusions were associated with a significantly ($P < 0.05$) increased risk of mortality, composite morbidity, pneumonia and sepsis [7]. These results were further demonstrated in a long-term review of 10,289 patients for up to 10 years post-coronary artery bypass surgery, in which RBC transfusion was associated with significant reductions in early (up to 6 months) and late (up to 10 years) survival when controlling for demographics, comorbidities, operative factors and early hazard for death [8].

ESAs are biological analogues of human erythropoietin produced in cell lines using recombinant DNA technology [9]. The major goals of ESA use are sustained correction of anaemia and resultant improvement in QoL for patients, while also reducing the need for RBC transfusions [4]. The first ESA was epoetin alfa; second and third generation ESAs have since been developed with a longer half-life than first-generation ESAs [10,11]. The impact of ESAs on overall patient survival and mortality in anaemia associated with cancer has been the subject of several trials and meta-analyses [12]. A large meta-analysis of 13,933 patients in 53 trials indicated that use of ESAs led to a reduction in survival and increased mortality in patients with cancer, with the authors recommending that the observed risks be measured against the benefits of ESA treatment [13]. However, while it cannot be ruled out, the same risk has yet to be confirmed in patients receiving chemotherapy [12].

Since the patent expiry of epoetin alfa, several biosimilar epoetins have become available [14]. Biosimilars, as defined by EU legislation, are therapeutic proteins exhibiting comparable quality, and non-clinical and clinical similarity to an existing reference biological medicine whose patent has expired. These biosimilars have undergone a rigorous preclinical, clinical and post-approval process to ensure that the mode of action, efficacy and safety are equivalent to originator products [15]. As part of this process, the Committee for Medicinal Products for Human Use (CHMP) recommends continuing post-approval studies of biosimilars to evaluate safety [16]. Once a product has been approved, such studies form part of an ongoing risk management plan through collection of real-life clinical evidence.

The present study, ORHEO (biOsimilaRs in the management of anaemia secondary to chemotherapy in HaEmatology and Oncology), was a post-marketing study aimed at observing Hb response in CIA patients presenting with various solid tumours, lymphoma or myeloma when treated with biosimilar epoetin alfa.

Methods

Study design

This was an observational, non-interventional, longitudinal, national, multicentre study (NCT02140736). After screening for inclusion and exclusion criteria, patients were enrolled in the study and followed-up for 6 months over three visits: inclusion visit (D0), 3-month follow-up visit (M3) and 6-month end-of-study visit (M6). The study protocol was reviewed and approved by the ethics committee (Advisory Committee on Information Processing Research in the Field of Health [Comité consultatif sur le traitement de l'information en matière de recherche dans le domaine de la santé], Ministry of Higher Education and Research, INSERM 700, Faculty of Medicine, Paris, France). Results were recorded on case report forms. As this is a post-approval, observational, non-interventional study only verbal consent was obtained in compliance with French law for such studies. Informed patient consent was obtained verbally prior to participation in the study and written consent was signed by the physician.

Patients

Inclusion criteria included patients at least 18 years of age presenting with CIA (irrespective of chemotherapy cycle) associated with solid tumours, lymphomas or myelomas and eligible for treatment with an epoetin alfa biosimilar.

Exclusion criteria included absence of chemotherapy treatment, presence of any contraindications for epoetin, hypersensitivity to any of the treatment components, previously confirmed pure red cell aplasia (PRCA), uncontrolled

hypertension, lack of access to anti-thrombotic prophylaxis, or involvement in another biosimilar epoetin study.

Primary and secondary objectives

The primary objective of the study was to observe response to treatment with an epoetin alfa biosimilar in patients presenting CIA in association with solid tumours, lymphoma or myeloma.

Secondary objectives included changes in biological indicators such as Hb, haematocrit, reticulocytes, serum iron, ferritin and blood pressure; disease outcome and the safety profile of epoetin alfa biosimilars. CIA was defined according to the World Health Organization (WHO) criteria [17], measured prior to starting treatment. A patient was described as a 'responder' to treatment with an epoetin biosimilar if Hb levels were at least equal to 10 g/dL; if there had been an increase in Hb levels of at least 1 g/dL since the inclusion visit; or if Hb level reached target level set by the physician on D0, without any blood transfusions in the 3 weeks prior to measurement (transfusion details were reported by investigators). In patients with baseline Hb levels at least equal to 10 g/dL, only those who reached their Hb target or had an Hb increase >1 g/dL were regarded as responders. Disease outcomes were reported using the WHO performance score [18].

Statistical methodology

All statistical analyses were performed on the per protocol population. This excluded patients with missing baseline Hb values, patients for whom age was not recorded, patients who had not initiated chemotherapy at baseline, patients who were not receiving an epoetin biosimilar at baseline and patients who switched to another epoetin treatment between baseline and Month 6. In addition, patients with a disease other than those described in the protocol were excluded. The rates of Hb responders were calculated at each visit with 95% confidence intervals, as well as logistic regressions for prognostic factors of Hb response, using SAS® software (9.2 release).

Results

Patient characteristics at baseline

A total of 2333 patients were included in the study (attended clinic at least once) from 235 investigational centres (see Figure 1). The rate of metastasis was 71.68% in the patient population with solid tumours. The main reasons for early withdrawal from the study were death of the patient (66.93%) followed by loss of contact (16.46%). All deaths were considered unrelated to treatment with epoetin biosimilars. Only 4.08% of patients withdrew early due to a change of ESA.

Demographic data are shown in Table 1. The mean baseline Hb level was 9.61 g/dL, across all patients. At baseline, 57.92% of patients had grade 1 anaemia (9.5–11 g/dL) and 35.58% of patients had grade 2 anaemia (8–9.5 g/dL). Target levels of 12–12.9 g/dL were set for 51.10 of patients and 10–11.9 g/dL set for 39.57% of patients. Overall, 16.75% of patients had at least one transfusion during

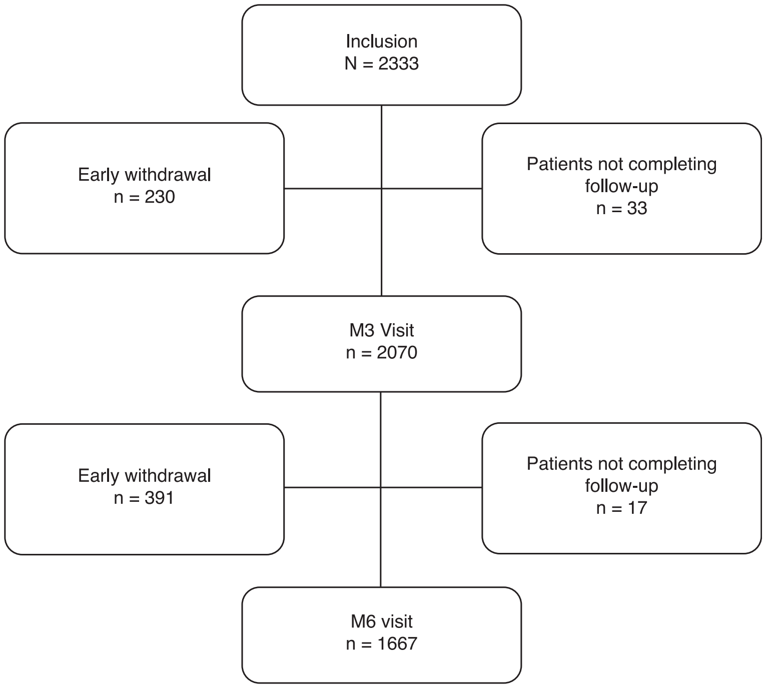

Figure 1 ORHEO study consort diagram.

Table 1 Patient demographics

Characteristic	Value
Age, mean ± SD (years)	66.49 ± 11.77
Patients with solid tumours, mean (years)	65.76
Patients with lymphoma, mean (years)	68.59
Patients with myeloma, mean (years)	70.65
Female (%)	48.6*
Male (%)	51.4*
Hb, mean ± SD (g/dL)	9.59 ± 0.88
sBP, mean ± SD (mmHg)	125.75 ± 14.48
dBP, mean ± SD (mmHg)	74.18 ± 9.79
Malignancies, n (%)	2310 (100)
Solid tumours, n (%)	1838 (79.57)
Lymphoma, n (%)	301 (13.03)
Myeloma, n (%)	171 (7.40)
Height, mean ± SD (cm)	166.35 ± 8.72
Weight, mean ± SD (kg)	67.46 ± 14.23
BMI, mean ± SD (kg/m^2)	24.33 ± 4.62

BMI, body mass index; dBP, diastolic blood pressure; Hb, haemoglobin; sBP, systolic blood pressure; SD, standard deviation.
*This table is corrected from the published version.

the year prior to inclusion. The majority (67.23%) of patients started their treatment with a biosimilar epoetin after inclusion, 3.90% of patients were already being treated with an epoetin biosimilar before inclusion, and the remainder began treatment on the date of inclusion.

Biosimilar epoetin treatment

Almost all (99.9%) patients received the biosimilar Retacrit® (epoetin zeta, Hospira); the remaining three patients received Binocrit® (epoetin alfa, Sandoz). The median dose of biosimilar epoetin was 30,000 IU/week during the course of the study. Most patients (99.7%) received biosimilar epoetin via the subcutaneous route and almost all (97.97%) received single weekly biosimilar epoetin injections.

Hb response

At M3, 81.6% (95% CI 79.91–83.26) of patients had responded to treatment compared to 86.5% at M6 across all disease categories (95% CI 84.80–88.10 [see Figure 2a]). The proportion of responders for patients with haematological malignancies (lymphoma and myeloma) and solid tumours were similar. Furthermore, the proportion of responders for two of the most commonly reported solid tumours was similar to the total study population. Of the patients with breast cancer, 86.80% (95% CI 82.01 –90.48) and 91.67% (95% CI 87.29–94.66) had responded to treatment at M3 and M6, respectively. Similarly, of the patients with lung cancer, 76.68% (95% CI 72.12–80.69) and 86.04% (95% CI 81.32–89.73) had responded to treatment at M3 and M6, respectively (see Figure 2b). Mean time to achievement of target Hb was 80.1 days for the responder population.

Overall, the mean change in Hb from inclusion to M3 was 1.52 ± 1.61 g/dL, compared to 1.72 ± 1.61 g/dL at M6 (See Figure 3a). An increase in Hb level of >2 g/dL was observed in 37% of patients at M3 and 44.2% of patients at M6. An increase in Hb of 1–2 g/dL was observed in 27.3% and 24.5% of patients at M3 and M6, respectively. Figure 3b shows the change in Hb level at M6, stratified by Hb level at study inclusion. Results were also analysed by disease type. In the solid tumour group, mean Hb levels increased by 1.44 g/dL and 1.65 g/dL at M3 and M6, respectively. In the lymphoma group, mean Hb increased by 1.72 g/dL and 2.12 g/dL at M3 and M6, respectively, while in the myeloma group, mean Hb increased by 2.03 g/dL and 1.57 g/dL, respectively. Where transfusion data were available, transfusion rates were 9.4% and 5.8% at M3 and M6, respectively for the total study population. Transfusion rates were 9.7% and 5.0% at M3 and M6, respectively for patients with solid tumours, 9.4% at both M3 and M6 for patients with lymphoma and 6.9% at both M3 and M6 for patients with myeloma.

Between inclusion and M6, a total of 1202 patients discontinued epoetin treatment. Of patients that discontinued, 39.5% of stops were due to satisfactory Hb being met, 26.6% were due to stopping or changing chemotherapy treatment, 14.9% were due to satisfactory Hb being reached and cessation of chemotherapy, 10.8% were due to inefficacy and 1.9% stopped due to an adverse effect.

Clinical parameters

Haematocrit, reticulocyte, iron, ferritin and blood pressure readings did not differ significantly at D0, M3 and M6 (see Table 2). A large standard deviation was observed in reticulocyte count. This could be explained by fluctuation in cell numbers as a result of chemotherapy cycles. Blood pressure was consistent across D0 (mean sBP: 125.75 mmHg, mean dBP: 74.18 mmHg), M3 (mean sBP: 125.58 mmHg, mean dBP: 74.51 mmHg) and M6 (mean sBP: 126.04 mmHg, mean dBP: 74.33 mmHg).

Safety

Overall, 17.1% of patients experienced at least one clinically significant adverse event (see Table 3). The rate of thromboembolic events was 2.4% and 1.5% at 3 and 6 months, respectively. During the course of the study, 12% of patients were treated with an antithrombotic agent (lymphoma: 9.3%; myeloma: 33.3%; solid tumour: 10.4%). A rise in blood pressure was reported in 1.3% of patients at M3. The same rate was reported at M6.

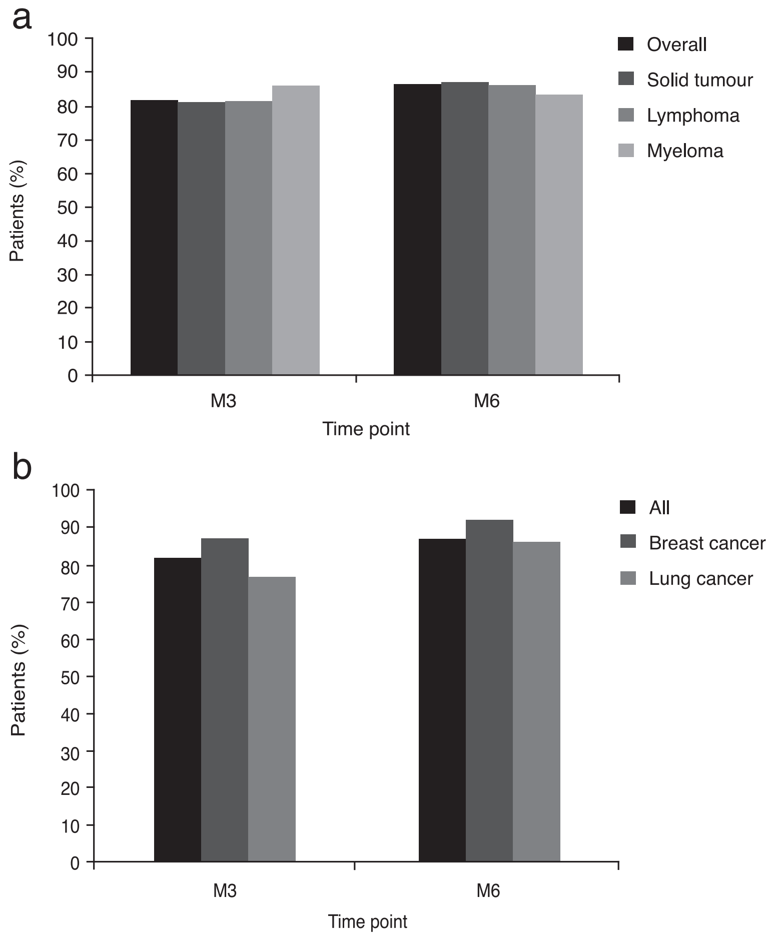

Figure 2 Treatment response stratified (a) by disease category and (b) by the most commonly reported solid tumours.

Overall treatment outcomes

At D0 73% of patients had a performance status of 0 or 1, compared with 68.3 and 69.7% of patients at M3 and M6, respectively (see Figure 4a).

Clinical progress was recorded as either 'exacerbation', 'stable' or 'improvement'. Improvement was seen in 40.20% and 40.38% of patients at M3 and M6, respectively (see Figure 4b).

Associated treatments

Associated treatments included antithrombotic agents (12% overall), IV iron (10.3% overall), oral iron (16.3%), folates (6.6% overall), vitamin B (4.3% overall) with a small proportion taking other vitamins (1.6% overall). A further 17.1% of patients were reported as being on 'other treatments'.

Discussion

Anaemia is a common complication of chemotherapy treatment. The advent of ESAs has led to a reduction in transfusion requirements, improved QoL and reduced fatigue for patients with CIA [2]. An important limitation of ESAs and biological medicines in general remains the high cost, which may limit access in some countries [19]. EU-approved biosimilar medicines can offer physicians the reassurance of rigorous comparability studies and extensive clinical and post-marketing surveillance programmes, in addition to cost savings due to lower development costs. Post-approval studies are an important aspect of post-approval efficacy and safety assessment of biosimilar medicines. This study forms part of an ongoing surveillance programme for epoetin zeta, a biosimilar of epoetin alfa.

The primary objective of this observational study was to record the rate of response to treatment of CIA with biosimilar epoetins in the oncology and onco-haematology settings. The definitions of Hb response utilised herein reflect the nature of this study, as it not only includes achieving target Hb but also clinically significant increases in Hb (achievement of 10 g/dL, or an increase in 1 g/dL from inclusion). The results of this study contribute to the growing body of evidence regarding the efficacy and safety of epoetin biosimilars in oncology.

The efficacy results reported herein correlate closely with previous research on epoetin zeta. A phase III

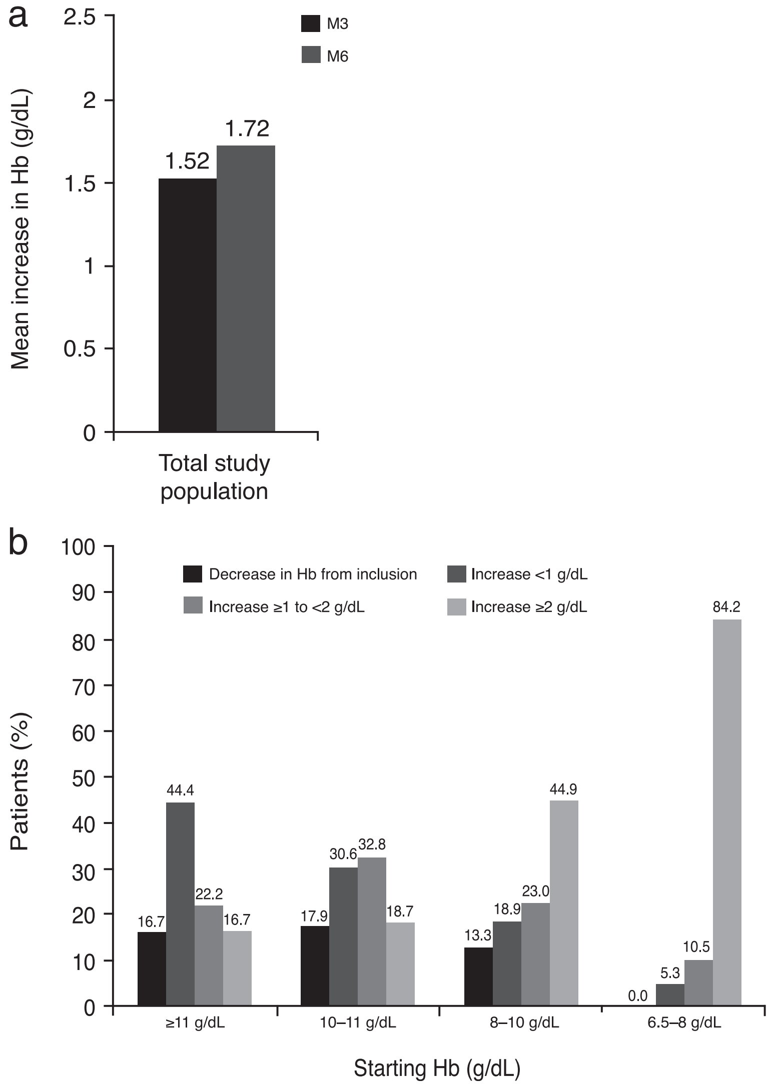

Figure 3 Mean change (a) from baseline in haemoglobin level in total study population and (b) change after six months stratified by baseline haemoglobin level.

non-controlled study of epoetin zeta in CIA over a 12-week period reported a mean Hb increase of 1.8 g/dL in the intention-to-treat (ITT) population, with 81.5% of patients achieving ≥1 g/dL Hb increase [20], both outcomes being similar to the findings here. This was the first open-label study on biosimilars conducted in Europe, and while more are currently planned or underway, ORHEO remains the largest with regard to patient numbers. Mean Hb response reported in the current study is similar to that reported for epoetin alfa in CIA associated with multiple myeloma [21].

Table 2 Secondary outcomes

	D0 (n)	M3 (n)	M6 (n)
Haematocrit (%)	29.54 ± 4.12 (2158)	34.41 ± 5.78 (1839)	34.90 ± 6.03 (1419)
Reticulocytes (/mm^3)	53083.10 ± 43772.32 (330)	57478.34 ± 47391.55 (228)	52803.71 ± 52301.40 (146)
Iron (μg/100 mL)	94.96 ± 129.22 (546)	140.39 ± 152.77 (295)	144.98 ± 152.96 (182)
Ferritin (ng/mL)	510.55 ± 629.61 (555)	512.83 ± 801.58 (301)	390.82 ± 466.91 (214)
sBP (mmHg)	125.75 ± 14.48 (1934)	125.58 ± 15.80 (1675)	126.04 ± 15.41 (1353)
dBP (mmHg)	74.18 ± 9.79 (1930)	74.51 ± 10.31 (1675)	74.33 ± 10.02 (1351)

dBP, diastolic blood pressure; sBP, systolic blood pressure.

Table 3 Clinically significant adverse events

	Solid tumours	Lymphoma	Myeloma	Overall
Thromboembolic events (%)	3.74	1.10	5.0	3.49
Bleeding (%)	2.64	1.47	1.25	2.38
Infection (%)	4.23	8.82	6.88	5.04
Local intolerability (%)	0.06	0.37	0.63	0.15
Rise in blood pressure (%)	2.33	2.21	1.25	2.23
Other (%)	9.14	7.72	8.75	8.92

The rate of Hb response showed a reduction at M6 for patients with myeloma compared to M3. Myeloma therapy is associated with a relatively high risk of deep vein thrombosis, particularly in the case of thalidomide-anthracycline regimens [22]. A possible reason for the reduction in Hb response noted here could be the setting of more conservative treatment targets due to thromboembolic risk associated with myeloma chemotherapy and the disease itself.

No unexpected adverse events (AEs) were seen during the course of this study. The overall rate of treatment-related AEs was similar to that reported previously for epoetin zeta (10.4%). The rate of thromboembolic events (3.49%) was lower than reported previously for epoetin zeta (4.2%) [20] and epoetin alfa (4.0%). Patient death was the main reason for discontinuing this study. No deaths were considered related to treatment, and the death rate in the current study can be attributed to the characteristics of the population, particularly the high rate of metastatic cancer at baseline.

As part of this study, WHO performance scale data and disease progression were collected to give context to Hb response rates reported. At baseline, 73.0% of patients

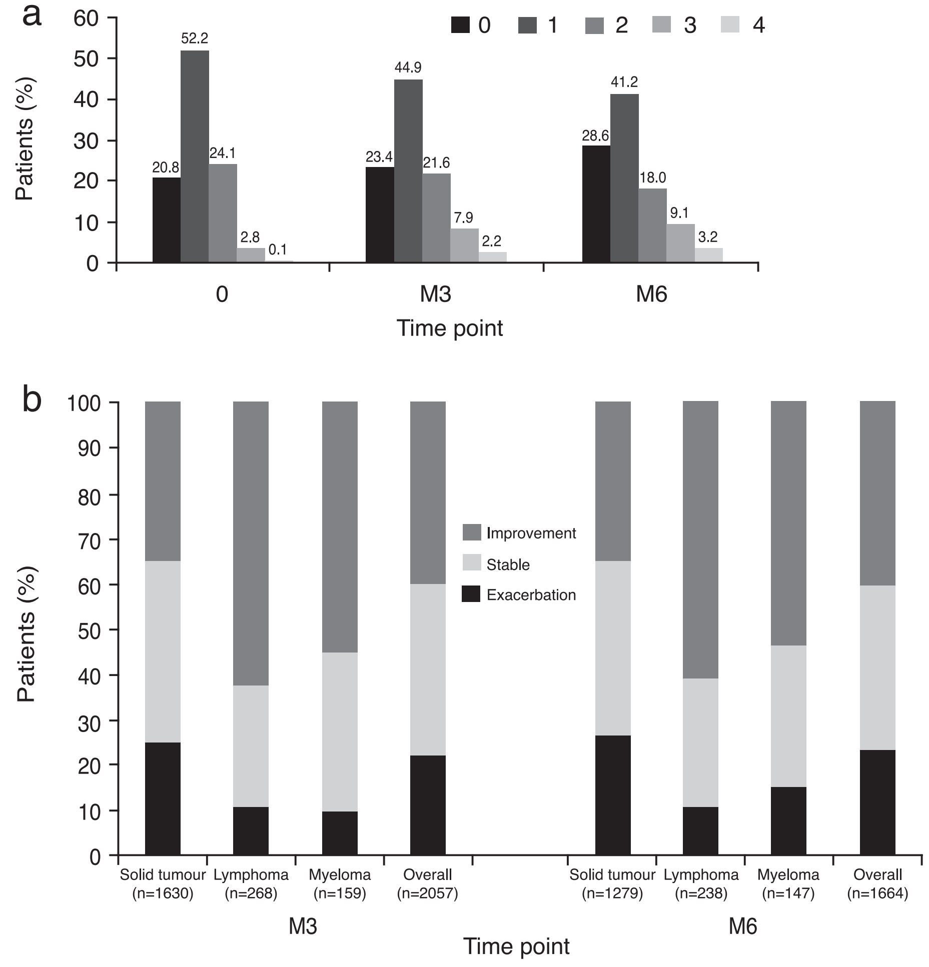

Figure 4 **Performance status (a) of all patients and (b) stratified by disease category.**

were characterised by a WHO performance status of 0 or 1, compared to 68.3% at M3 and 69.7% at M6. Evidence suggests Hb levels correlate with WHO performance status [23], and while it is hard to distinguish changes due to disease progression and changes due to improvement in Hb, the number of patients with a WHO performance status of 0 increased at both M3 and M6, indicating that some patients felt more physically able upon completion of the study. In terms of disease progression, an improvement in disease state was considerably more common in patients with a lymphoma, at M6 (60.9%). This could be a result of the efficacy/safety profiles of treatments in this group. Any improvement or exacerbation in disease outcomes should not be considered related to epoetin treatment. Such outcomes are confounded by patient characteristics such as age and disease status, as well as the nature of chemotherapy regimens, which vary considerably, particularly in the case of the solid tumour category.

This manuscript reports the results of an observational study. It is therefore difficult to address potential confounding factors surrounding treatment. However, it is anticipated that the ORHEO study can complement the findings of controlled clinical trials through offering real-life treatment observation. There was a significant disparity between the numbers of patients with solid tumours compared to those with myeloma or lymphoma, which corresponds well with the relative epidemiology of these cancer types in Europe [24,25]. The definition of response in this study relates to clinically meaningful change in Hb, as befits the real-life setting and observational nature. As such, the rate of response should not be compared with that reported in randomised controlled clinical trials. One important limitation in this study was the lack of representation from biosimilar epoetins other than Retacrit. This reflects availability at the centres involved during the course of the study, and is not through deliberate omission or inherent study design.

Given the negative impact anaemia has on the QoL and overall survival in most cancer types [2,3], it is not surprising that European guidelines recommend ESA treatment to correct CIA and reduce the need for transfusion [26]. Guidance from the EMA states that in patients with non-myeloid malignancies treated with chemotherapy and an Hb level of <10 g/dL, treatment with ESAs might be considered to increase Hb to ≤12 g/dL or to prevent further decline in Hb. In patients treated with curative intent, ESAs should be used with caution. Furthermore, the use of ESAs should be carefully reconsidered in patients with a high risk of thromboembolic events. ESAs have been of great use to vast numbers of cancer patients receiving chemotherapy and are associated with patient benefits such as freedom from RBC transfusion and improvements in QoL. However, biological medicines such as recombinant ESAs are costly. The introduction of biosimilar medicines has the potential to provide cost competition and reduce cost to the payer due to their lower development costs [27]. Indeed, a recent cost-efficiency study conducted in France, Germany, Italy, Spain, and the UK compared various regimens of biosimilar and originator ESAs in the management of CIA [19]. Biosimilar ESA therapy was consistently cost-efficient over treatment with originator products under both fixed and weight-based dosing scenarios. The cost benefits of biosimilar ESAs may therefore increase patient access to this important form of treatment. However, physicians should not be obliged to prescribe biosimilars for purely cost reasons; only on proof of quality, efficacy and safety should biosimilars be considered a viable option. This study adds to the body of evidence supporting the efficacy and safety of the biosimilar ESA epoetin zeta in patients with CIA.

Conclusions

This study, the largest of its type, demonstrated a high rate of response to epoetin zeta treatment in a large patient population with CIA. The rate of thromboembolic events was lower than previously observed for this treatment, while no unexpected treatment-related AEs were identified.

Observational studies present a valuable means of assessing efficacy and safety under clinical conditions and complement the current body of data derived from randomised controlled trials.

This study assessed the clinical profile of biosimilar epoetin zeta as part of an ongoing commitment to post-marketing observation, and demonstrated that epoetin zeta is well-tolerated and efficacious in treating anaemia in cancer patients with CIA.

Competing interests

EL, NAA, VB, DL, SN, BN, PN, RR and NS have declared no conflict of interest. MM declares research support from Novartis, Pfizer, Astellas, MSD and Genzyme, has participated in an advisory panel for Hospira Ltd and has acted as a consultant for Bristol-Meyers Squibb. PS acts as a board member for Roche, Celgene, and Mundipharma. FM declares research support from Pfizer, Amgen and Roche. He has also participated in advisory boards and received research support from Novartis Hospira and Bristol-Meyers Squibb. JLL and JLM have received research support from Hospira. LMD declares research support from Hospira, Teva and Amgen and that he has acted as a consultant for Roche. MC declared research support from Sandoz, Roche, Teva and Octapharma. HA is an employee of Hospira France.

Authors' contributions

All authors had full access to the data and had final responsibility for the decision to submit the manuscript. MM, EL, PS and HA performed the research, collected the data, participated in the research design, contributed essential reagents and tools and wrote and reviewed the manuscript. NAA, VB, MC, LMD, JLL, DL, FM, JLM, SN, BN, PN, RR and NS collected the data and reviewed the manuscript. All authors read and approved the final manuscript.

Acknowledgements

The authors gratefully acknowledge the patients and clinical investigators who participated in this trial. Medical writing and editorial assistance was

provided by Dr Marion James, apothecom scopemedical ltd, funded by Hospira Ltd. Statistical analyses were performed by Clinact, funded by Hospira Ltd.

Funding

This trial was funded by Hospira SAS France.

Author details

[1]Service d'Hématologie, Pavillon Marcel Bérard 1G, Centre Hospitalier Lyon Sud, 165 Chemin du Grand Revoyet, Lyon- Pierre Bénite 69495, France. [2]Centre Alexis Vautrin, Vandoeuvre-les-Nancy, France. [3]Institut Bergonie, Bordeaux, France. [4]Centre Hospitalier de Troyes, Troyes, France. [5]Centre Hospitalier de Carcassonne, Carcassonne, France. [6]Centre Hospitalier de Montauban, Montauban, France. [7]Clinique Sainte Anne, Strasbourg, France. [8]Polyclinique Montréal, Carcassonne, France. [9]Clinique Pasteur, Evreux, France. [10]Clinique Rambot-Provencale, Aix en Provence, France. [11]Centre Hospitalier d'Aix en Provence, Aix en Provence, France. [12]Centre Hospitalier Régional Universitaire de Tours, Tours, France. [13]Clinique du Cap d'Or, La Seyne sur Mer, France. [14]Centre Frédéric JOLLIOT, Rouen, France. [15]Centre Hospitalier de Montbéliard, Montbeliard, France. [16]Laboratoire HOSPIRA France, Meudon La Foret, France.

Received: 4 February 2014 Accepted: 1 July 2014
Published: 10 July 2014

References

1. Ludwig H, Van Belle S, Barrett-Lee P, Birgegård G, Bokemeyer C, Gascón P, Kosmidis P, Krzakowski M, Nortier J, Olmi P, Schneider M, Schrijvers D: **The European Cancer Anaemia Survey (ECAS): a large, multinational, prospective survey defining the prevalence, incidence, and treatment of anaemia in cancer patients.** *Eur J Cancer* 2004, **40:**2293–2306.
2. Cella D, Kallich J, McDermott A, Xu X: **The longitudinal relationship of hemoglobin, fatigue and quality of life in anemic cancer patients: results from five randomized clinical trials.** *Ann Oncol* 2004, **15:**979–986.
3. Caro JJ, Salas M, Ward A, Goss G: **Anemia as an independent prognostic factor for survival in patients with cancer: a systemic, quantitative review.** *Cancer* 2001, **91:**2214–2221.
4. Aapro MS: **Anemia management with erythropoiesis-stimulating agents: a risk-benefit update.** *Oncologist* 2008, **13**(Suppl 3):1–3.
5. Ludwig H: **Anemia of hematologic malignancies: what are the treatment options?** *Semin Oncol* 2002, **29**(Suppl 8):45–54.
6. Stramer SL: **Current risks of transfusion-transmitted agents: a review.** *Arch Pat Lab Med* 2007, **131:**702–707.
7. Bernard AC, Davenport DL, Chang PK, Vaughan TB, Zwischenberger JB: **Intraoperative transfusion of 1 U to 2 U packed red blood cells is associated with increased 30-day mortality, surgical-site infection, pneumonia, and sepsis in general surgery patients.** *J Am Coll Surg* 2009, **208:**931–937.
8. Koch CG, Li L, Duncan AI, Mihaljevic T, Loop FD, Starr NJ, Blackstone EH: **Transfusion in coronary artery bypass grafting is associated with reduced long-term survival.** *Ann Thorac Surg* 2006, **81:**1650–1657.
9. Macdougall IC: **Novel erythropoiesis-stimulating agents: a new era in anemia management.** *Clin J Am Soc Nephrol* 2008, **3:**200–207.
10. Topf JM: **CERA: third-generation erythropoiesis-stimulating agent.** *Expert Opin Pharmacother* 2008, **9:**839–849.
11. Wauters I, Vansteenkiste J: **Darbepoetin alfa in the treatment of chemotherapy-induced anaemia.** *Expert Opin Biol Ther* 2009, **9:**221–230.
12. Bohlius J, Tonia T, Schwarzer G: **Twist and shout: one decade of meta-analyses of erythropoiesis-stimulating agents in cancer patients.** *Acta Haematol* 2011, **125:**55–67.
13. Bohlius J, Schmidlin K, Brillant C, Schwarzer G, Trelle S, Seidenfeld J, Zwahlen M, Clarke M, Weingart O, Kluge S, Piper M, Rades D, Steensma DP, Djulbegovic B, Fey MF, Ray-Coquard I, Machtay M, Moebus V, Thomas G, Untch M, Schumacher M, Egger M, Engert A: **Recombinant human erythropoiesis-stimulating agents and mortality in patients with cancer: a meta-analysis of randomised trials.** *Lancet* 2009, **373:**1532–1542.
14. Brinks V, Hawe A, Basmeleh AH, Joachin-Rodriguez L, Haselberg R, Somsen GW, Jiskoot W, Schellekens H: **Quality of original and biosimilar epoetin products.** *Pharm Res* 2011, **28:**386–393.
15. European Generic Medicines Association: *Biosimilars Handbook.* 2nd edition. London: Sage Publications Ltd; 2011.
16. CHMP: **Guideline on similar biological medicinal products containing biotechnology-derived proteins as active substance: non-clinical and clinical issues.** http://www.ema.europa.eu/docs/en_GB/document_library/Scientific_guideline/2009/09/WC500003920.pdf.
17. Groopman JE, Itri LM: **Chemotherapy-induced anemia in adults: incidence and treatment.** *J Nat Cancer Inst* 1999, **91:**1616–1634.
18. Oken MM, Creech RH, Tormey DC, Horton J, Davis TE, McFadden ET, Carbone PP: **Toxicity and response criteria of the Eastern Cooperative Oncology Group.** *Am J Clin Oncol* 1982, **5:**649–655.
19. Aapro M, Cornes P, Sun D, Abraham I: **Comparative cost efficiency across the European G5 countries of originators and a biosimilar erythropoiesis-stimulating agent to manage chemotherapy-induced anemia in patients with cancer.** *Ther Adv Med Oncol* 2012, **4:**95–105.
20. Tzekova V, Mihaylov G, Elezovic I, Koytchev R: **Therapeutic effects of epoetin zeta in the treatment of chemotherapy-induced anaemia.** *Curr Med Res Opin* 2009, **25:**1689–1697.
21. Dammacco F, Castoldi G, Rödjer S: **Efficacy of epoetin alfa in the treatment of anaemia of multiple myeloma.** *Br J Haematol* 2001, **113:**172–179.
22. Zonder JA: **Thrombotic complications of myeloma therapy.** *Hematology Am Soc Hematol Educ Program* 2006: 348–355.
23. Birgegård G, Gascón P, Ludwig H: **Evaluation of anaemia in patients with multiple myeloma and lymphoma: findings of the European CANCER ANAEMIA SURVEY.** *Eur J Haematol* 2006, **77:**378–386.
24. Sant M, Allemani C, Tereanu C, De Angelis R, Capocaccia R, Visser O, Marcos-Gragera R, Maynadié M, Simonetti A, Lutz JM, Berrino F: **Incidence of hematologic malignancies in Europe by morphologic subtype: results of the HAEMACARE project.** *Blood* 2010, **116:**3724–3734.
25. Ferlay J, Autier P, Boniol M, Heanue M, Colombet M, Boyle P: **Estimates of the cancer incidence and mortality in Europe in 2006.** *Ann Oncol* 2007, **18:**581–592.
26. Schrijvers D, De Samblanx H, Roila F: **Erythropoiesis-stimulating agents in the treatment of anaemia in cancer patients: ESMO Clinical Practice Guidelines for use.** *Ann Oncol* 2010, **21**(Suppl. 5):v244–v247.
27. Zelenetz AD, Ahmed I, Braud EL, Cross JD, Davenport-Ennis N, Dickinson BD, Goldberg SE, Gottlieb S, Johnson PE, Lyman GH, Markus R, Matulonis UA, Reinke D, Li EC, DeMartino J, Larsen JK, Hoffman JM: **NCCN Biosimilars white paper: regulatory, scientific, and patient safety perspectives.** *J Natl Compr Canc Netw* 2011, **9**(Suppl. 4):S1–S22.

doi:10.1186/1471-2407-14-503
Cite this article as: Michallet *et al.*: BiOsimilaRs in the management of anaemia secondary to chemotherapy in HaEmatology and Oncology: results of the ORHEO observational study. *BMC Cancer* 2014 **14**:503.

Submit your next manuscript to BioMed Central and take full advantage of:

- **Convenient online submission**
- **Thorough peer review**
- **No space constraints or color figure charges**
- **Immediate publication on acceptance**
- **Inclusion in PubMed, CAS, Scopus and Google Scholar**
- **Research which is freely available for redistribution**

Submit your manuscript at
www.biomedcentral.com/submit

BioMed Central
